# A Rare Case Report of Persistent Hiccups as an Atypical Presentation of COVID-19

**DOI:** 10.7759/cureus.13625

**Published:** 2021-03-01

**Authors:** Raed Atiyat, Sindhusha Veeraballi, Neveen Al-Atiyat, Kok Hoe Chan, Jihad Slim

**Affiliations:** 1 Medical Education, Saint Michael's Medical Center, Newark, USA; 2 Medical Education, American University of Antigua, Antigua, ATG; 3 Internal Medicine, Saint Michael's Medical Center, Newark, USA; 4 Infectious Diseases, Saint Michael's Medical Center, Newark, USA

**Keywords:** hiccups, spasm of diaphragm, covid-19, sars-cov-2

## Abstract

Sudden, involuntary spasm of the diaphragm associated with closure of the glottis will lead to a hiccup sound. Brief episodes of hiccups are often self-limiting and may be physiologically encountered in everyday life. However, prolonged attacks of hiccups are associated with significant morbidity. Herein, we present a rare and interesting case of coronavirus disease 2019 (COVID-19) induced persistent hiccups in a 61-year-old gentleman with no signs or symptoms of gastric pathology. As the patient required less oxygen supplementation, with inflammatory markers down-trending, his hiccups improved. The timeline of the symptom presentation and his response to treatment highly suggested that his hiccups were associated with COVID-19 infection. To the best of our knowledge, there are only two cases of persistent hiccups secondary to COVID-19 that have been reported thus far. Our case will add to the existing literature and highlight the potential association of COVID-19 with hiccups.

## Introduction

Severe acute respiratory syndrome coronavirus 2 (SARS-CoV-2) is a single-stranded ribonucleic acid virus that binds to angiotensin-converting enzyme 2 receptors (ACE-2), which are abundantly present throughout the human body and not localized to a single organ [1,2]. ACE-2 receptors are found on the epithelial cells of the lung, small intestine, vascular endothelium, and various other organs [1,2]. Thus, SARS-CoV-2 has the potential to cause a very large variety of symptoms that might be underreported and misdiagnosed.

The medical term of hiccups is singultus, which is derived from the Latin word “Singult” meaning the act of catching one's breathe while sobbing. Sudden contraction of diaphragmatic muscle and intercostal muscles causes a rapid rush of air into lungs, leading to abrupt closure of the glottis and thereby producing a sound “hic” [3,4]. Hiccups are recurrent involuntary contractions of the diaphragm. The diaphragm and inspiratory muscles make an uncontrolled strong contraction to shift the diaphragm downward, causing a sudden inspiration of a lot of air [5]. This causes a sudden increase in pressure that is counteracted with the glottis closing, thus causing the hiccup sound [5]. An underreported atypical presentation in patients with coronavirus disease 2019 (COVID-19) is persistent hiccups lasting more than 48 hours. Normally hiccups are brief and resolve spontaneously. Chronic or persistent hiccup can be an indication of an underlying illness [5]. To the best of our knowledge, there are only two case reports with persistent hiccups as atypical presentation of COVID-19 in the literature.

## Case presentation

A 61-year-old gentleman with a past medical history of hypertension presented to our hospital with a one-week history of intermittent sharp mid-sternal chest pain radiating to the back and subjective fevers. In addition, he complained of persistent hiccups for two days. Otherwise, he denied cough, dyspnea, loss of smell, taste, chills, headaches, palpitation, abdominal pain, nausea, vomiting, changes in urinary or bowel habits, sick contacts, or any recent travels. He had no prior history of hiccups, hiatal hernia, hysteria, or any gastrointestinal pathologies. He smokes two cigarettes daily but denied any alcohol or illicit drug use. His only medication was hydrochlorothiazide.

**Figure 1:**
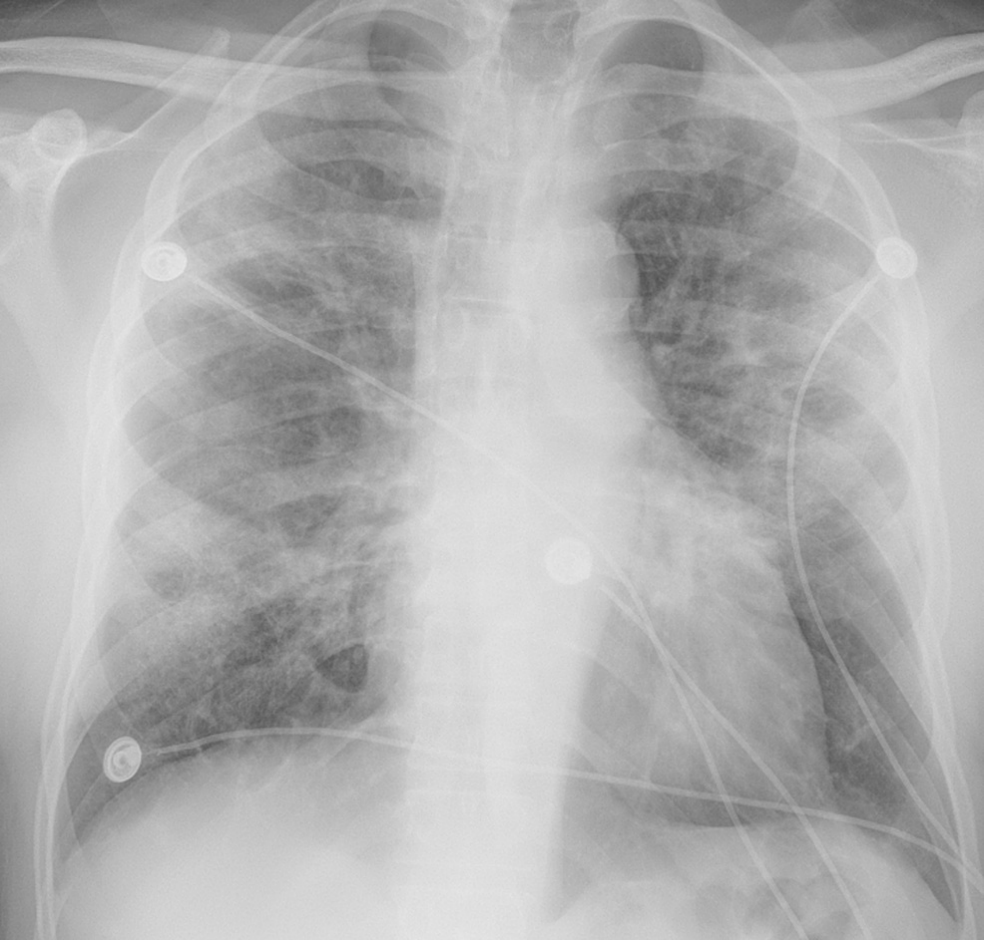
Chest X-ray displayed bilateral mid-lung opacities, with the right greater than the left, consistent with multifocal pneumonia

Initial vitals showed a temperature of 97.5°F, heart rate of 89 beats/min, respiratory rate of 17 breaths/min, blood pressure of 155/77 mmHg, and saturating 97% on room air. Physical examination was notable for non-reproducible chest pain, no murmurs, rubs, or gallops appreciated. Pulmonary examination revealed diffuse bilateral wheezes with no rales or rhonchi. Electrocardiogram (EKG) showed normal sinus rhythm. Troponins were negative. Chest X-ray displayed bilateral mid lung opacities, with the right greater than the left, consistent with multifocal pneumonia (Figure 1).

Inflammatory markers were all elevated: D-dimer was 1,047 (normal range < 500), ferritin was 1,480 ng/mL (normal range: 24-336 ng/mL), LDH (lactate dehydrogenase) was 433 U/L (normal range: 122-222 U/L), and CRP (C-reactive protein) was 22.9 mg/dL (normal range: 0-0.8 mg/dL), along with lactic acid of 2.3 mmol/L (normal range: 0-2 mmol/L) and procalcitonin of 5.3 ng/mL (normal range: 0-0.5 ng/mL). Liver function test, renal function test, calcium, and electrolytes were all within normal limits. Blood cultures, rapid influenza test, *Legionella* Ag, and *Mycoplasma pneumoniae* antibody were all negative. His nasopharyngeal SARS-CoV-2 RT-PCR (reverse transcriptase-polymerase chain reaction) was positive.

The patient received one dose of ceftriaxone and was started on azithromycin. He also received pantoprazole 40 mg daily for stress ulcer prophylaxis. While in the medical unit, the patient began to desaturate; he was then placed on oxygen supplementation and started on Decadron® 6 mg daily. On the third day of presentation, the patient’s hiccups were so debilitating that his oxygen saturation decreased to the low eighties, his face became flushed, and he had difficulty in eating and breathing. His hiccups were partially relieved with metoclopramide, which was given as needed. As the patient required less oxygen supplementation, with inflammatory markers down-trending, the hiccups improved.

## Discussion

Hiccups that are usually self-limiting are very common in everyday life. However, hiccups when prolonged can be associated with significant morbidity. Hiccups can be triggered by anything that irritates the diaphragm and phrenic nerve such as electrolyte disturbances, uremia, peritonitis, renal insufficiency, bronchitis, pericarditis, gastroenteritis, cholecystitis, asthma, anxiety, foreign body in ear, pneumonia, and myocardial infarction [3,4].

Herein, our patient had persistent hiccups, which was only partially relieved by metoclopramide. All the potential triggering factors were ruled out. Hiccups resolved four days after he started treatment for COVID-19 and showed clinical improvement. On literature review, we found two case reports highlighting the potential association of hiccups with COVID-19 presentation [1,6]. The first case was of a 62-year-old man who presented with weight loss and hiccups. All other potential causes were ruled out and he tested positive for COVID-19 [1]. The second case was of a 48-year-old man who presented with sore throat and hiccups and was found to have COVID-19. His hiccups resolved with Baclofen® 15 mg three times daily [6]. The limitation of both those case reports is the possible coincidence of hiccups with COVID-19 infection. Herein, we presented this case with similar presentation to strengthen the idea that hiccups could be an atypical presentation of COVID-19.

The exact pathogenesis of hiccups in COVID-19 are poorly understood; it may be cause by stress gastritis or hepatitis with irritation of the diaphragm. It could also be due to renal insufficiency, uremia, or electrolytes abnormalities leading to hiccups. Nonetheless, none of this was present in our patient. Our patient had normal liver function test, renal function, and electrolytes. Our patient also had no pneumonia (workups for pneumonia were negative), pericarditis, or myocardial infarction. Moreover, the hiccups did not improve with chlorpromazine and metoclopramide, but, in fact, resolved when the oxygenation improved and inflammatory markers started to down-trend. This highlight the potential role of inflammation or cytokine storm in the pathogenesis of persistent hiccups observed in patients with COVID-19.

SARS-CoV-2 infection presented with several atypical extra-pulmonary presentations such as diarrhea, abdominal pain, confusion, headache, abnormal lab results with no clinical symptoms, conjunctivitis, isolated anosmia, dysgeusia, pernio-like acral lesions, and others. There is continuous evolution of knowledge on COVID-19 during this dreadful pandemic. We emphasize that the persistent hiccups can be an atypical presentation of COVID-19, and more awareness is necessary among physicians to consider COVID-19 as one of the differentials in patients with persistent hiccups. It is important to take necessary contact precautions in such cases to prevent the spread of this dreadful virus. More investigations are needed in order to further understand the physiology and pathogenesis of hiccups in COVID-19.

## Conclusions

COVID-19 has a multifaceted presentation. Persistent hiccups could be an atypical presentation of COVID-19. Our case report would like to highlight this rare entity and hopefully able to raise the awareness among physicians regarding this rare presentation.

## References

[1] Prince G, Sergel M (2020). Persistent hiccups as an atypical presenting complaint of COVID-19. Am J Emerg Med.

[2] Metzger R, Franke FE, Bohle RM, Alhenc-Gelas F, Danilov SM (2011). Heterogeneous distribution of angiotensin I-converting enzyme (CD143) in the human and rat vascular systems: vessel, organ and species specificity. Microvasc Res.

[3] Launois S, Bizec JL, Whitelaw WA, Cabane J, Derenne JP (1993). Hiccup in adults: an overview. Eur Respir J.

[4] Federspil PA, Zenk J (1999). Singultus [Hiccup]. HNO.

[5] Chang FY, Lu CL (2012). Hiccup: mystery, nature and treatment. J Neurogastroenterol Motil.

[6] Bakheet N, Fouad R, Kassem AM, Hussin W, El-Shazly M (2021). Persistent hiccup: a rare presentation of COVID-19. Respir Investig.

